# Association of TCF4 polymorphisms and fuchs’ endothelial dystrophy: a meta-analysis

**DOI:** 10.1186/s12886-015-0055-6

**Published:** 2015-06-19

**Authors:** Dan Li, XiaoYan Peng, HuiYu Sun

**Affiliations:** Beijng Tongren Eye Center, Beijing Ophthalmology and Visual Sciences Key Lab, Beijing Tongren Hospital, Capital Medical University, 1 Dong Jiao Min Xiang, Dong Cheng District, Beijing, 100730 China; Department of Ophthalmology, Beijng di tan Hospital, Capital Medical University, Beijing, China

## Abstract

**Background:**

Studies investigating the associations between *transcription factor 4 (TCF4)* genetic polymorphisms and Fuchs’ endothelial dystrophy (FED) have reported controversial results. Therefore, this meta-analysis aims to clarify the effects of *TCF4* polymorphisms on FED risk.

**Methods:**

A meta-analysis was conducted to assess the association between four single nucleotide polymorphisms (SNPs) in*TCF4* and the risk of FED. Relevant studies were selected through an extensive search of PubMed, EMBASE, and the Web of Science databases. Pooled odds ratio (OR) and 95 % confidence interval (CI) were calculated using the random-effects model.

**Results:**

Thirteen studies were included in this systematic review and meta-analysis. The pooled results showed that there was a strong positive association between the *TCF4* rs613872 polymorphism and FED risk in all the genetic models tested (G allele vs. T allele: OR = 4.19, 95 % CI = 3.53–4.97; GG vs. GT/TT: OR = 4.27, 95 % CI = 2.54–7.19; GG/GT vs. TT: OR = 6.29, 95 % CI = 4.23–8.93; GG VS. TT: OR = 10.64, 95 % CI = 5.28–21.41; GT VS. TT: OR = 6.08, 95 % CI = 4.28–8.64). Statistic evidence was also detected for a significant association between three other SNPs and the risk of FED.

**Conclusions:**

This meta-analysis suggested a genetic association between four *TCF4* polymorphisms (rs613872, rs2286812, rs17595731, and rs9954153) and the risk of FED.

## Background

Fuchs’ endothelial dystrophy (FED) is a familial, slowly progressive, and irreversible disorder affecting the corneal endothelial cell monolayer [1]. It has been reported that, in the United States, FED affects about 4 % of the population over the age of forty [2], making it the most common genetic disorder of the corneal endothelium. The most important signs of FED are the thickening of Descemet’s membrane and microscopic collagenous excrescences known asguttae [3]. Disease progression results in reduced vision as a result of the edema in the cornea, which is caused by loss of the fluid-pumping function of the endothelium [4].

Although the precise reason for FED remains unclear, recent studies have reported significant insights into the genetic basis of the disorder. To date, several gene mutations, such as *collagen type 8 alpha-2 (COL8A2), the sodium borate cotransporter gene (SLC4A11), transcription factor 8 (TCF8)*, and *transcription factor 4 (TCF4),* have been implicated in the pathogenesis of FED [5–8]. Of these gene mutations, the *TCF4* variations have been considered to be associated with a number of diseases, such as FED, schizophrenia, and primary sclerosing cholangitis [8–10]. The *TCF4* gene is located on chromosome 18q [11]. It encodes a transcription factor protein, E2-2, which is a member of the E protein family that is expressed in the cornea during development and which is involved in regulating cellular growth and differentiation [12].

Recently, *TCF4* polymorphisms have attracted a lot of attention. A previous genome-wide association study (GWAS) showed a significant relationship between FED and four genotyped single nucleotide polymorphisms (SNPs) (rs17595731, rs613872, rs9954153, and rs2286812) of the *TCF4* gene [8]. Since then, several case–control studies have also been conducted and they have also shown that the *TCF4* polymorphisms are associated with FED [8, 13–16]. However, these results remain inconclusive. For example, Nanda et al. [17] reported that the *TCF4* rs613872 polymorphism was not associated with FED, but Kuot et al. [11] found that the *TCF4* rs613872 polymorphism was a risk factor for FED. Other SNPs of *TCF4* (rs2286812, rs17595731, and rs9954153) were also analyzed in some FED genetic association studies.

Meta-analysis is a powerful statistical technique that is often used in combination with different studies; therefore, it draws a more comprehensive conclusion. With this in mind, we conducted a meta-analysis to summarize all the relevant evidence for an association between the risk of FED and genetic polymorphisms of *TCF4*, which include four SNPs: rs17595731, rs613872, rs9954153, and rs2286812.

## Methods

### Literature search

Literature searches were performed in PubMed (http://www.ncbi.nlm.nih.gov/pubmed/), ISI Web of Science (www.webofknowledge.com), and EMBASE (http://www.embase.com) databases. Key search terms included (“Fuchs’ endothelial dystrophy,” or “Fuchs’ endothelial corneal dystrophy,”) and (“*transcription factor 4*,” or “*TCF4*,”or “*immunoglobulin transcription factor 2,*” or “E2-2,” or “SL3-3 enhancer factor 2,” or “SEF2,” or “rs613872,” “rs17595731” or “rs2286812” OR “rs9954153”) and (“polymorphism,” or “variation,” or “mutation,” or “variant,” or “genotype,” or “allele”). References cited in each eligible literature were manual checked until no further studies were found. If the overlapping patient population was included in several studies, the latest study was included. The final literature search was updated on April 05, 2015, with no restrictions as to publication year, language, or methodological filter.

### Inclusion and exclusion criteria

Studies included in this meta-analysis were required to meet the following inclusion criteria: (a) designed as nested case–control, case–control, or GWAS; (b) evaluated *TCF4* polymorphism and FED; (c) odds ratio (OR) and the corresponding 95 % confidence interval (CI) were provided; and (d) sufficient genotypic or allelic information was provided to estimate. Exclusion criteria were: (a) case-only studies, familial studies, or duplicate data; (b) abstracts, comments, letters, reviews, or editorial articles; (c) insufficient genotyping data.

### Data extraction and quality assessment

Two observers (L.D. and S.H.Y.) independently extracted the following information from included studies, using a standardized data extraction form: first author, year of publication, country of origin, population ethnicity, source of controls, numbers of genotyped cases and controls, and Hardy-Weinberg equilibrium (HWE) for the control group. If a study provided several risk estimates, the best adjusted estimate was extracted. Any disagreement was resolved by discussion or adjudicated with the involvement of a third reviewer (P.X.Y.). The quality of each study was assessed using the Newcastle-Ottawa Scale (NOS) [18]. The NOS uses a “star” rating system to judge quality, based on three aspects of the study: selection, comparability, and exposure; scores range from 0 stars (worst) to 9 stars (best). Studies with a score of ≥ 7 were considered of high quality [19]. Any discrepancies were addressed by means of discussion and consensus.

### Statistical analyses

ORs and 95 % CIs were used to assess the strength of the associations between *TCF4* polymorphisms and FED. Adjusted ORs and 95%CIs were used if they were reported; otherwise, the pooled ORs and 95 % CIs without adjustments were calculated for the following genotypic models for rs613872 SNP: allele (G vs. T); homozygote (GG vs. TT), heterozygote (GT vs. TT), dominant (GG/GT vs. TT), and recessive (GG vs. GT/TT). Due of the insufficient data for the other three *TCF4* SNPs, the pooled ORs and the 95 % CIs were calculated using an additive model. Data were combined using a random effects model to achieve more conservative estimates. Statistical heterogeneity between the studies was evaluated using Cochran’s Q test and the I^2^ statistic. For the Q statistic, *p* < 0.05 was considered to indicate statistically significant heterogeneity [20]. I^2^ was also used to assess the heterogeneity in the meta-analysis, and heterogeneity was said to exist when I^2^ > 50 % [21]. To determine the reliability of the outcomes of the meta-analysis, a sensitivity analysis was performed by the exclusion of an individual study each time. Furthermore, we repeated the sensitivity analyses to delete the findings from studies that deviated from the HWE principle [22] and to calculate the pooled ORs for the remainder of the studies [8, 13–17, 23–25]. Finally, to detect publication biases, Begg’s and Egger’s measures were calculated and assessed using Begg’s funnel plots [26, 27]. A *p* value less than 0.05 was considered statistically significant in the test for overall effect. The analysis was conducted using the Stata software package (Version 12.0; Stata Corp., College Station, TX, USA).

## Results

### Literature search and characteristics

**Fig. 1:**
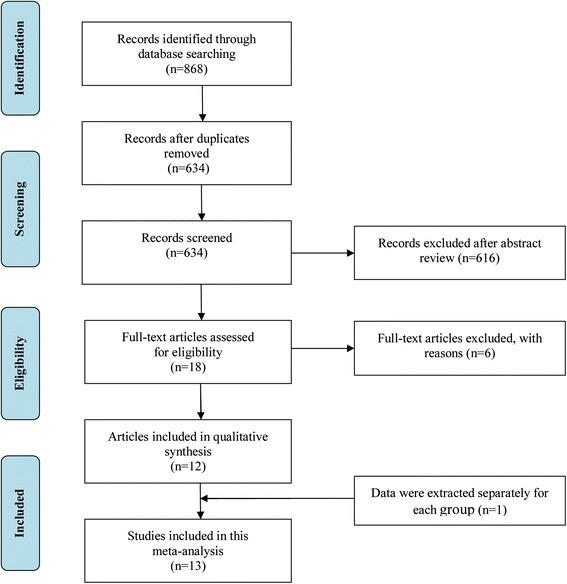
Flow diagram outlining the selection process for inclusion of studies in the systematic review and meta-analysis

The initial search yielded 868 potentially relevant studies. After the removal of duplicates through electronic databases, 634 studies remained. Based on titles and abstracts, 616 articles were excluded because of their apparent irrelevance. In total, 18 full-text articles were further assessed for eligibility. Of these, six articles were excluded for the following reasons: the article was a review (n = 1) [28]; the articles lacked controls (n = 3) [29–31]; and the articles did not focus on the relative polymorphism (n = 2) [32, 33]. Finally, 12 articles met the inclusion criteria and were included in this meta-analysis [8, 13–17, 22–25, 34, 35]. One trial [8], reported the allele and the genotype of the discovery group and the replication group, respectively. We assumed that the discovery group and the replication group were the subjects in two separate studies. Overall, 13 studies were included in this meta-analysis. Of these, 11 studies reported the association between the *TCF4* rs613872 polymorphism and FED. Three studies reported the association between the *TCF4* rs9954153 polymorphism and FED. Three studies reported the association between the *TCF4* rs17595731 polymorphism and FED. Five studies reported the association between the *TCF4* rs2286812 polymorphism and FED. The study selection process is detailed in Fig. 1. The main characteristics of the included studies are presented in Table 1. Among these studies, nine originated from the United States, one from Australia, one from India, one from China, and one from Singapore. Thirteen studies included 2468 FED cases and 2902 controls. The NOS results showed that the average score was 7.9 (range: 7 to 9), indicating that the methodological quality was generally good.

**Table 1:** Characteristics of included studies

First Author (year)	Country	Ethnicity	Study design	Genotyping method	Case/Control	Source of control	SNP	HW-E test
Baratz (2010)^a^ [8]	USA	Caucasian	GWAS	Taqman assay	130/260	HB	rs613872,rs17595731,	yes
							rs9954153,rs2286812	
Baratz (2010)^b^ [8]	USA	Caucasian	GWAS	Taqman assay	150/150	HB	rs613872,rs17595731,	yes
							rs9954153,rs2286812	
Li (2011) [23]	USA	Caucasian	Case–control	Taqman assay	450/340	HB	rs613872	yes
Riazuddin (2011) [24]	USA	Caucasian	Case–control	Taqman assay	170/180	HB	rs613872	yes
Thalamuthu (2011) [34]	Singapore	Asian	Case–control	MassArray	57/121	HB	rs2286812	yes
Igo (2012) [22]	USA	Caucasian	Case–control	Taqman assay	531/204	HB	rs613872	no
Kuot (2012) [16]	Australia	Caucasian	Case–control	MassArray	103/275	HB	rs613872,rs9954153,	yes
							rs2286812,rs17595731	
Stamler (2013) [15]	USA	Caucasian	Case–control	Taqman assay	82/163	HB	rs613872	yes
Nanda (2014) [17]	India	Asian	Case–control	PCR-sequencing	44/108	HB	rs613872	yes
Wang (2014) [35]	China	Asian	Case–control	Taqman assay	34/491	HB	rs2286812	yes
Li (2014) [19]	USA	Caucasian	Case–control	Taqman assay	529/494	HB	rs613872	yes
Mootha (2014) [13]	USA	Caucasian	Case–control	Taqman assay	120/100	HB	rs613872	yes
Wieben (2014) [25]	USA	Caucasian	Case–control	PCR-sequencing	68/16	HB	rs613872	yes

*HW-E* Hardy Weinberg equilibrium; *PCR* polymerase chain reaction; *HB* hospital-based

^a^The data extracted from discovery group

^b^The data extracted from replication group

### Meta-analysis of *TCF4* rs613872polymorphism and FED

**Fig. 2:**
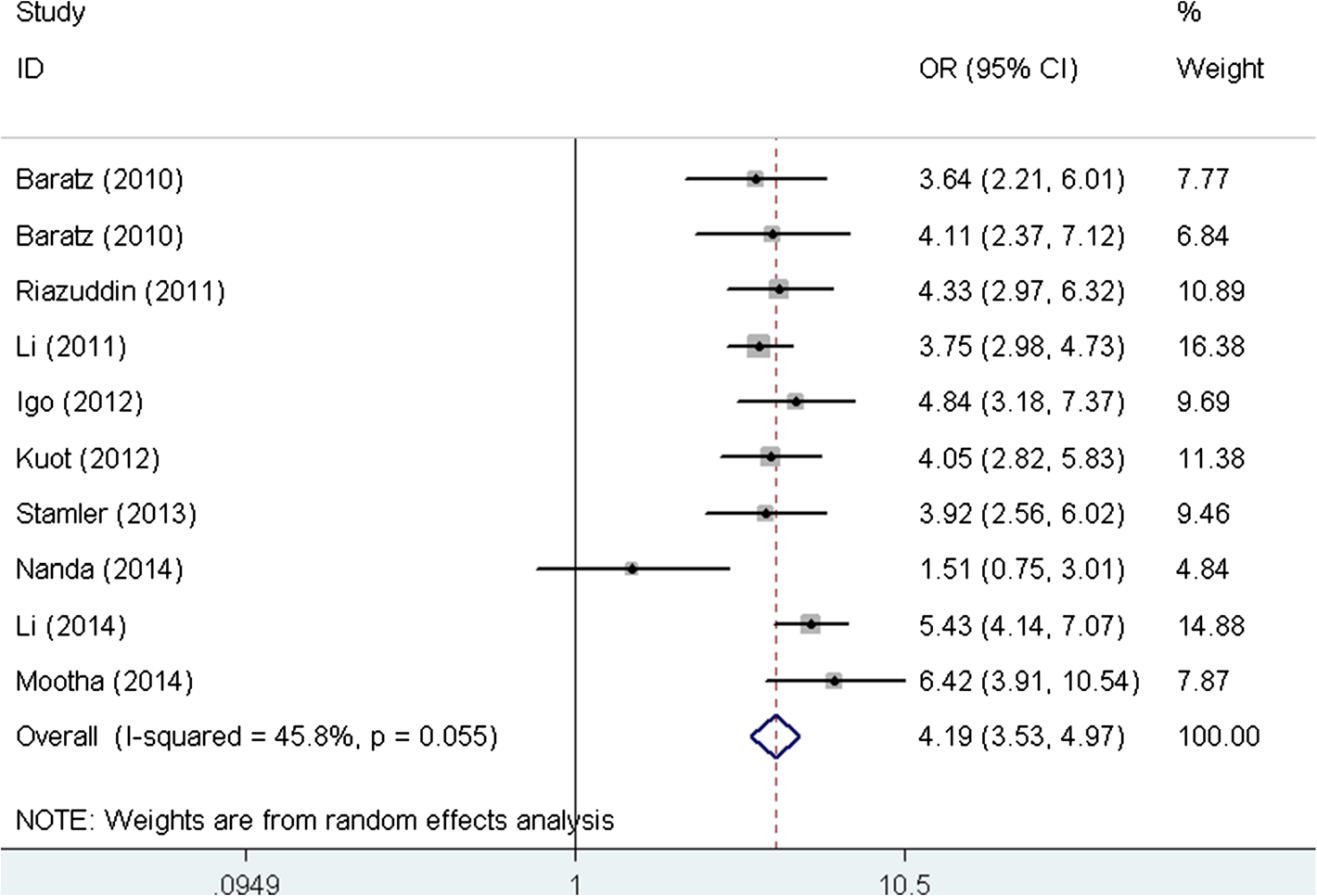
Forest plot for the association between *TCF4* rs613872 polymorphism and FED risk (G allele vs. T allele). OR: odds ratio; CI: confidence interval; *TCF4*: transcription factor 4; FED: Fuchs endothelial dystrophy

Table 2 presents the main results of the pooled ORs and the heterogeneity test of the meta-analysis. For rs613872, the pooled ORs showed that there was a strong positive association between the *TCF4* rs613872 polymorphism and FED risk in all the genetic models tested (G allele vs. T allele: OR = 4.19, 95 % CI = 3.53–4.97 (Fig. 2); GG vs. GT/TT: OR = 4.27, 95 % CI = 2.54–7.19; GG/GT vs. TT: OR = 6.29, 95 % CI = 4.23–8.93; GG vs.TT: OR = 10.64, 95 % CI = 5.28–21.41; GT vs. TT: OR = 6.08, 95 % CI = 4.28–8.64). Among the studies, significant heterogeneity was detected in the dominant model (GG/GT vs. TT) and the heterozygote model (GT vs. TT) (Table 2). For rs2286812, five studies were included for calculation and the pooled ORs and 95%CI was 1.77(1.19–2.63) in the additive model, which also showed a genetic association with the risk of FED. Significant associations were also observed for rs17595731 and rs9954153 (Table 2).

**Table 2:** Results of meta-analysis for TCF4 polymorphisms and risk of FED

Polymorphism (comparison)	No. of studies	OR (95%CI)	*P*	Heterogeneity			*P* Egger’s test^a^	*P* Begg’s test^b^
				*x* ^2^	I^2^	*P*		
rs613872								
G vs. T	10	4.19(3.53–4.97)	<0.001	16.60	45.8 %	0.055	0.411	0.474
GG vs. GT/TT	6	4.27(2.54–7.19)	<0.001	6.20	19.3 %	0.288	0.295	0.260
GG/GT vs. TT	7	6.29(4.23–8.93)	<0.001	16.23	63.0 %	0.013	0.586	0.707
GG VS. TT	6	10.64(5.28–21.41)	<0.001	9.43	47.0 %	0.093	0.378	0.452
GT VS. TT	6	6.08 (4.28–8.64)	<0.001	12.72	60.7 %	0.026	0.938	0.707
rs2286812								
T vs C	5	1.77(1.19–2.63)	0.005	12.10	66.9 %	0.017	0.207	0.180
rs17595731								
C vs G	3	4.70(3.06–7.21)	<0.001	1.27	0.0 %	0.530	0.775	1.000
rs9954153								
G vs T	3	2.43(1.97–3.01)	<0.001	0.20	0.0 %	0.906	0.793	1.000

*TCF4* transcription factor 4; *FED* Fuchs endothelial dystrophy; *OR* odds ratio; *CI* confidence Interval

^a^ *P* Egger’s test = the *P* value for Egger’s test

^b^ *P* Begg’s test = the *P* value for Begg’s test

### Sensitivity analysis and publication bias

**Fig. 3:**
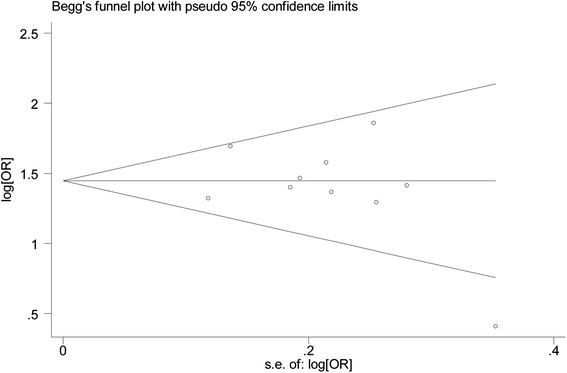
Funnel plot for studies of the association of *TCF4* rs613872 polymorphism and FED risk (G allele vs. T allele). OR: odds ratio; *TCF4*: transcription factor 4; FED: Fuchs endothelial dystrophy

To evaluate the influence of an individual data set on the pooled results, one study was deleted at a time. The estimates were not altered substantially when any single study was deleted, suggesting the high stability of the meta-analysis results for *TCF4* rs613872 (Table 3). This analysis of the association between the*TCF4* rs613872 polymorphism and FED risk also revealed that one study by Nanda et al. [17] was the main origin of heterogeneity (Table 3). The I^2^ significantly declined from 45.8 % to 0.0 % (Q = 7.69, p = 0.464) after removing the study by Nanda et al. It is interesting to note that when one study that deviated from the HWE principle was deleted [22], the results from the remaining studies were similar to the overall result, and a significant association was detected in all the genetic models. We also performed “leave-one-out” sensitivity analyses for the other three SNPs and the results showed that no single study influenced the pooled results (data not shown). Publication bias was estimated using Begg’s funnel plot and Egger’s test (Table 2). No significant publication bias was observed in this meta-analysis. In addition, the funnel plot for studies of the association between the*TCF4* rs613872 polymorphism and FED risk under an additive model (G allele vs. T allele) is presented in Fig. 3.

**Table 3:** Sensitivity analysis of the meta-analysis results for TCF4 rs613872 polymorphism and FED risk (G allele vs. T allele)

	Random effects model		Test of homogeneity		
Study Excluded	OR	95%CI	Q	I^2^ (%)	*P*-value
None	4.19	3.53–4.97	16.60	45.8	0.055
Baratz (2010)^a^ [8]	4.23	3.52–5.09	16.22	50.7	0.039
Baratz (2010)^b^ [8]	4.19	3.48–5.04	16.58	51.8	0.035
Li (2011) [23]	4.26	3.50–5.19	15.12	47.1	0.057
Riazuddin (2011) [24]	4.16	3.43–5.04	16.59	51.8	0.035
Igo (2012) [22]	4.11	3.41–4.96	16.20	50.6	0.040
Kuot (2012) [16]	4.19	3.46–5.09	16.53	51.6	0.035
Stamler (2013) [15]	4.21	3.48–5.08	16.46	51.4	0.036
Nanda (2014) [17]	4.38	3.89–4.94	7.69	0.0	0.464
Li (2014) [19]	4.01	3.37–4.78	12.57	51.4	0.042
Mootha (2014) [13]	4.05	3.42–4.80	13.78	41.9	0.088

*TCF4* transcription factor 4; *FED* Fuchs’ endothelial dystrophy; *OR* odds ratio; *CI* confidence Interval

^a^The data extracted from discovery group

^b^The data extracted from replication group

## Discussion

Despite the fact that the GWAS publication noted that genetic variations in *TCF4* polymorphisms contribute to the development of FED, this correlation also needs to be verified by a case control study. Therefore, we performed this meta-analysis to provide the highest level of evidence for the association between *TCF4* polymorphisms and the risk of FED. With a total of 5370 participants, this meta-analysis focused on the association between *TCF4* variants and the risk of FED, thirteen studies addressing one or more SNPs in *TCF4* (rs613872, rs17595731, rs9954153, and rs2286812) were included. The pooled results showed that a significant association was detected between FED risk and the rs17595731, rs613872, rs9954153, and rs2286812 SNPs. This s consistent with the findings presented in the GWAS publication [8]. For rs613872, in order to achieve robust and reliable results for the meta-analysis of the association between rs613872 and FED risk, we performed a series of analyses. These sensitivity analyses were performed by excluding one individual study each time. This procedure did not greatly change the pooled results; rather, it supported their reliability. In addition, it should be noted that the genetic distributions of the controls in one study deviated from HWE, indicating the possibility of bias. Thus, we conducted the sensitivity analyses again, omitting that study. The results from the pooled ORs before and after omitting the studies that deviated from the studies that used the HWE principles were similar, suggesting that the results were minimally affected by this study. In addition, for the other SNPs, no individual study influenced the pooled results Furthermore, no significant publication bias was observed in the pooled results in all four of the SNPs, further demonstrating the robustness of our meta-analysis.

Differences in ethnic groups may affect genetic predisposition to human diseases [36, 37]. In the present meta-analysis, we found that only one study from India did not detect the association between the *TCF4* rs613872 polymorphism and the risk of FED, which might be due to the different ethnicity of the included study participants [17]. Another study from Asian also showed no association between rs2286812 and the risk of FED, which also might be due to the different ethnicity of the study population. However, only one study with a small sample size should be considered. In the future, more epidemiologic studies of people with Asian ethnicity using larger sample sizes are needed to further confirm this difference.

To date, the pathomechanism underlying the association between the *TCF4* gene and FED risk is still unclear. Possible mechanisms for the association between the *TCF4* gene and FED risk have been proposed. First, the protein produced by the *TCF4* gene (E2-2) might participate in endothelium growth, proliferation, and differentiation [38]. Therefore, loss of function in this protein through gene mutations would reduce the number of endothelial cells, as observed in FED patients [8]. In addition, the E2-2 protein has been shown to up-regulate the protein expression of zinc finger E-box binding homeobox 1 (ZEB1), which has been found to be the pathogenic protein of FED. Thus, this indirectly showed that the *TCF4* gene mutation associated with FED results from changes in the expression of ZEB1 [8]. Taken together, these proofs supported the hypothesis that the *TCF4* gene might heighten the risk of FED.

An important aim of meta-analysis is to determine the sources of heterogeneity. In this meta-analysis, substantial heterogeneity was observed in the dominant model (I^2^ = 63.0 %; p = 0.013), and the heterozygote model (I^2^ = 60.7 %; p = 0.026) for rs613872. We performed the sensitivity analysis by exclusion of one individual study each time and we found that the study by Nanda et al. [17] might be the source of heterogeneity, as heterogeneity was significantly decreased after excluding that study. For rs2286812, substantial heterogeneity was also observed in the additive model and the sensitivity analysis also showed that Wang’s [35] study is the source of the heterogeneity.

In performing a quantitative analysis of the relationship between *TCF4* polymorphisms and FED risk, this meta-analysis was also limited. First, the number of original studies included in the meta-analysis was relatively small; for rs17595731, rs9954153, and rs2286812, only three to five studies were included. Second, substantial heterogeneity was observed among the studies. However, we determined that the source of this heterogeneity was the single study from India for rs613872. When this study was excluded, the results of the remaining studies did not change, and the heterogeneity was greatly reduced. Third, the genotyping methods differed among these studies, which may have affected the results. Fourth, it should be noted that the HWE test was not performed in one of the studies, which may have increased selection bias in the control. However, when that study was excluded the summary OR was unchanged in the remaining studies, which suggested the high stability of the results of this meta-analysis.

## Conclusions

In conclusion, this meta-analysis suggested a genetic association between four *TCF4* polymorphisms (rs613872, rs2286812, rs17595731, and rs9954153) and the risk of FED. Despite these encouraging findings, the inherent limitations of the studies should be considered, and conclusions drawn from the pooled results should be interpreted with caution. In the future, more epidemiologic studies of other ethnicities with a well-designed and larger sample size are needed to further confirm our findings.
